# Expression patterns of *TRα* and *CRABPII* genes in Chinese cashmere goat skin during prenatal development

**DOI:** 10.1186/s40781-015-0060-6

**Published:** 2015-08-20

**Authors:** Tao Zhong, Wei Zhao, Zhongqiang Zhou, Li Li, Linjie Wang, Hua Li, Hongping Zhang

**Affiliations:** Farm Animal Genetic Resources Exploration and Innovation Key Laboratory of Sichuan Province, College of Animal Science and Technology, Sichuan Agricultural University, Chengdu, Sichuan 611130 P. R. China; School of Life Science, Foshan University, Foshan, 528000 P. R. China

**Keywords:** Cashmere goat, *TRα*, *CRABPII*, Skin, Expression

## Abstract

**Background:**

The physiologic characteristics of the cashmere trait and many of the differentially expressed genes relevant to hair cycling have been extensively studied, whereas genes involved in the prenatal development of hair follicles have been poorly investigated in cashmere goats. The aim of this study, therefore, was to quantify the time-course changes in the expressions of *TRα* and *CRABPII* genes in the fetal skin of Chinese cashmere goats at the multiple embryonic days (E70, E75, E80, E90, E100, E120 and E130) using real-time quantitative PCR (RT-qPCR).

**Results:**

RT-qPCR showed that *TRα* was expressed at E70 with relatively high level and then slightly decreased (E75, E80, and E90). The highest expression of *TRα* mRNA was revealed at E130 (*P* > 0.05). The expression pattern of *CRABPII* mRNA showed an ‘up-down-up’ trend, which revealed a significantly highest expression at E75 (*P* < 0.05) and was down-regulated during E80 to E120 (*P* < 0.05) and mildly increased at E130, subsequently.

**Conclusion:**

This study demonstrated that *TRα* and *CRABPII* genes expressed in different levels during prenatal development of cashmere. The present study will be helpful to provide the comprehensive understanding of *TRα* and *CRABPII* genes expressions during cashmere formation and lay the ground for further studies on their roles in regulation of cashmere growth in goats.

**Electronic supplementary material:**

The online version of this article (doi:10.1186/s40781-015-0060-6) contains supplementary material, which is available to authorized users.

## Background

The Inner Mongolian cashmere goat is a Chinese indigenous breed characterized as a double-coated species. The outer coat consists of coarse guard hairs and the undercoat is the soft and precious cashmere. Two kinds of hair follicles which known as primary hair follicles and secondary hair follicles existed in the skin of the Inner Mongolian cashmere goat. Cashmere, which is derived from the secondary hair follicles, has smaller diameters than wool fibers produced by the primary hair follicles. Primary hair follicles and secondary hair follicles form at different periods and play different roles in the development of hair. In mice, the primary hair follicles arose *in utero* from embryonic day (E) 12.5 and the secondary follicles started to develop until E17 [1, 2]. In goat embryos, the precursor primary follicles were observed in head, neck, shoulder, and belly at E45. The hair follicles gradually formed during 55E to 65E and developed into the mature primary follicles at E135 [3]. The morphogenesis and development of the secondary follicles were similar to those of the primary follicles. The secondary follicles grew from E65 to E75 and then extended to skin surface. The complete structure of the secondary follicle was formed at E135 in the embryos of Chinese cashmere goats [4]. Furthermore, the periodic growth of the secondary follicles also presented in a breed-specific manner [5]. All primary follicles but few secondary follicles were mature at birth and the number of secondary follicles increased 10-fold in the 57 days after birth. The number of primary follicles showed a tendency to decline between 57 and 107 days of age in Australian cashmere goats [6]. Like the hair follicle cycling in other mammals, the growth of cashmere in goats was also tightly programmed by the three synchronized interchanging stages, anagen (growth phase), catagen (regression phase) and telogen (resting phase) throughout postnatal life [7, 8].

Some genes involved in the growth and development of hair follicles in cashmere goats have been identified, such as *KAPs* [9, 10], *BMP* [11], *Prolactin* [12], and *Keratin* [13, 14]. Furthermore, in mammals, the thyroid hormones (TH) have multi-functions in many important physiological processes including the normal growth, development, differentiation and metabolism. Recently, new insights into TH biological function have been obtained from animal studies involving in epidermis, dermis and hair cycling including anagen prolongation and stimulation of both hair matrix keratinocyte proliferation and hair pigmentation [15, 16]. The metabolism of TH is related to deiodinase, which is also regulated cashmere growth by altering its activity in skin tissue [17, 18]. TH action could be mediated through the thyroid hormone receptor (TR), which is part of the nuclear hormone receptor superfamily and bound to TH in three patterns identified in skin [19, 20]. TR interacts with the hairless gene product, a transcription factor required for hair growth. TR has been detected in epidermal keratinocytes, skin fibroblasts and a number of cell types that made up the hair follicles. In addition, the retinoic acid (RA) is essential for the development and maintenance of hair cycling [21]. The cellular RA-binding protein type II (CRABPII) is involved in RA synthesis pathway, which could shuttle RA to its receptor in nucleus and increase its transcriptional efficiency [22]. Therefore, the dynamic expression of *CRABPII* mRNA could affect the concentration of RA, which acts on the formation of cashmere though regulating sebaceous gland. During hair cycle, the expression pattern of the RA synthesis and signaling including *Crbp*, *Dhrs9*, *Aldh1a1*, *Aldh1a2*, *Aldh1a3* and *Crabp2* defined in rodents only [23].

Based on the genetic studies in humans and rodents, *TRα* and *CRABPII* acted important roles in driving the progression of the hair cycle. We postulate that these two genes might have functions during cashmere formation in goat. So in this study, we described the characteristics of *TRα* and *CRABPII* genes in the Inner Mongolian cashmere goat and identified their expression patterns in skin tissue during the middle late embryonic stages (E70 to E130).

## Materials and methods

### Animal and skin tissue preparation

The Inner Mongolia cashmere goat is a traditional outstanding breed, which is famous for its excellent cashmere performance and strong adaptation to the semi-desert and desert steppes. The tested individuals were selected from the Aerbasi White Cashmere Goat Breeding Farm in Inner Mongolia Province, China. Twenty-one embryos (three samples at each stage) were randomly collected and any lineage was avoided during the sampling process. Skin samples (approximately 1 cm^2^ for each individual) were collected from right mid-side of embryos at seven different embryonic days (E70, E75, E80, E90, E100, E120 and E130). Tissue was frozen in liquid nitrogen and stored at −80 °C for further analysis. All the experimental procedures for this experiment were conducted under a protocol approved by the Institutional Animal Care and Use Committee in the College of Animal Science and Technology, Sichuan Agricultural University, China.

### RNA isolation and cDNA synthesis

The frozen skin tissues were ground using mortars in liquid nitrogen and the total RNA was isolated by Trizol reagent (Invitrogen, Carlsbad CA, USA) according to the manufacturer's protocols. The concentration and quality of the total RNA were further assessed using the NanoDrop spectrophotometer (Bio-Rad, Benicia, USA). The RNase-free DNase I (Promega, Madison, USA) was used to digest genomic DNA. The first-strand cDNA was synthesized using the M-MLV reverse transcriptase kit (Promega, Madison, USA) with oligo (dT) primer.

### Gene cloning and quantitative PCR analysis

Three primer pairs were designed to amplify the caprine *TRα* and *CRABPII* genes according to their conserved regions of homologies from human, mouse, cattle, sheep and pig (Table 1). PCR was carried out in a 25 μL reaction mixture containing 2 μL first-strand cDNA, 50 mM KCl, 10 mM Tris–HCl (pH 8.3), 0.5 mM MgCl_2_, 10 pmol each primer, 150 μM dNTPs and 1 unit *Taq* polymerase (TaKaRa, Dalian, China). The cycling condition included an initial denaturation step at 95 °C for 5 min, 38 cycles of at 94 °C for 30 s, annealing temperature for 30 s and extension at 72 °C for 45 s, and a final extension at 72 °C for 7 min in a PTC-100 PCR thermocycler (MJ Research, Inc., Watertown, MA). PCR products were ligated with the pMD19-T vector (TaKaRa, Dalian, China) after purification, and sequenced by Invitrogen Biotech Co. Ltd. (Shanghai, China).

**Table 1:** The RT-PCR and qRT-PCR primers used in this study

Primer Name	Sequence (5’-3’)	Fragment size (bp)	T.M. (°C)
*Cloning primers*			
*TRα-*1 F	CCTGGATGGAATTGAAGTGA	799	62.0
*TRα-*1R	GACATGATCTCCATGCAGC		
*TRα-*2 F	AGGCCTTCAGCGAGTTTAC	652	59.0
*TRα-*2R	CCTTCTCTCCAGGCTCCTC		
*CRABPII*-1 F	CAGTGCTCCAGTGGAAAGA	563	56.5
*CRABPII*-1R	CCAGAAGTGATTGGGTGAG		
*Real-time PCR primers*			
*TRα-*3 F	TTACCTGGACAAAGACGAGC	113	57.4
*TRα-*3R	TCTGGATTGTGCGGCGAAAG		
*CRABPII*-2 F	ACATCAAAACCTCCACCACC	111	56.5
*CRABPII*-2R	CCCATTTCACCAGGCTCTTA		
*ACTB-*F	CCTGCGGCATTCACGAAACTAC	87	58.5
*ACTB-*R	ACAGCACCGTGTTGGCGTAGAG		
*GAPDH-*F	GCA AGTTCCACGGCACAG	249	59.0
*GAPDH-*R	GGT TCACGCCCATCACAA		
*TOP2B-*F	GTGTGGAGCCTGAGTGGTATA	137	59.0
*TOP2B-*R	AAGCATTCGCCTGACATTGTT		

The quantitative PCR (qRT-PCR) was carried out in an iCycler iQ Real-Time PCR Detection System (Bio-Rad, Benicia, USA) with a total volume of 20 μL containing 10 μL 2 × SYBR Premix Ex Taq II, 0.6 μL primers (10 μM) and 1 μL diluted cDNA. PCR reaction was as follows: a 95 °C denaturation for 30 s, followed by 40 cycles of 94 °C for 15 s, annealing temperature for 30 s, and 72 °C for 30 s. A melting program ranging from 55 °C to 95 °C with a heating rate of 0.5 °C/10 s was carried out to create the melt curves. Reactions were performed in triplicate and negative control was also performed in parallel.

### Normalization of the expression data

In the present study, three internal control genes (*ACTB*, *GAPDH* and *TOP2B*, Table 1) were selected to normalize the expression levels of *TRα* and *CRABPII* mRNAs. To accurate expression profiling of target genes, the geometric mean of multiple carefully selected housekeeping genes was validated as an accurate normalization factor [24]. The relative gene expression was calculated with the 2^-ΔΔCt^ method [25]. Data were presented as mean ± SE. Comparisons between groups were analyzed *via* GLM (General Linear Model) for experiments with more than 2 subgroups. The significance level was *P* < 0.05.

## Results and discussion

### Characteristics of goat *TRα* and *CRABPII* mRNAs

**Fig. 1:**
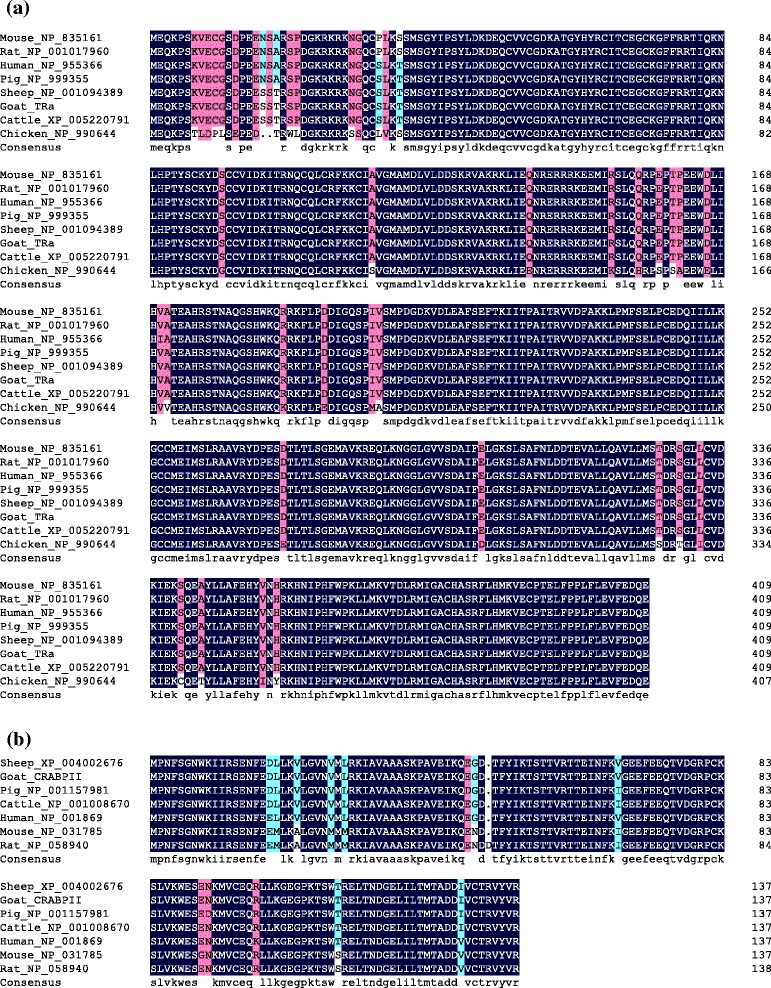
Alignment of the *TRα* (**a**) and *CRABPII* (**b**) amino acid sequences

A 1,309-bp fragment of *TRα* was assembled by the two overlapped sequences of *TRα*-1 F/1R and *TRα*-2 F/2R with an open reading frame (ORF) extending from nucleotide positions 21 to 1,253 (with reference to the translational start codon of ATG), which encoded a protein with 410 amino acids (Accession No. KF589923). The obtained sequence of *CRABPII* mRNA was 563 bp in length with an ORF of 417 bp encoding 138 amino acids (Accession No. KF589924). The blast results revealed that both of *TRα* and *CRABPII* were quite conserved among species (Fig. 1, Additional file 1: Figure S1 and Additional file 2: Figure S2). The sequence similarity ranged from 88 % to 100 % (Additional file 3: Table S1). The coding sequence of the caprine *TRα* gene shows a high similarity with the sequences in other mammals, sharing 99 % identity with sheep (NM_001100919) and cattle (NM_001046329). The goat *CRABPII* shows 88 % identity with mice (NM_007759) and 98 % identity with cattle (NM_001008670).

**Fig. 2:**
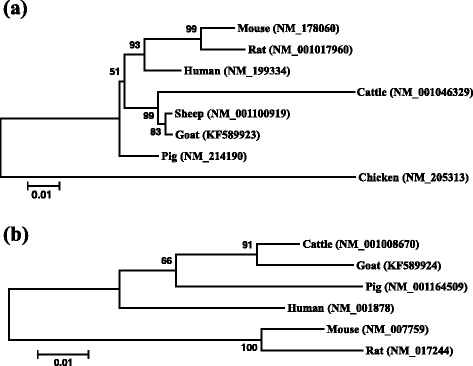
The phylogenetic trees constructed by coding sequences of *TRα* (**a**) and *CRABPII* (**b**) based on the Neighbor-Joining method

The nucleotide sequences were aligned by the Cluster W method included in the program BioEdit version 7.2.5 [26]. The phylogenetic analysis was constructed using the program MEGA 4.1 [27], with a Kimura 2-parameter model and a bootstrap test (1000 replications). The phylogenetic tree revealed that the goat *TR* grouped with sheep, and then clustered with cattle, pig, human, mice and chicken subsequently (Fig. 2a). The phylogenetic tree of *CRABPII* gene showed a similar clustering with differences in sort of branch-length groups (Fig. 2b). The Minimum Evolution, Maximum Parsimony and UPGMA trees revealed the same clustering groups as presented by the NJ trees (data not shown).

### Time-course expressions of *TRα* and *CRABPII* genes

**Fig. 3:**
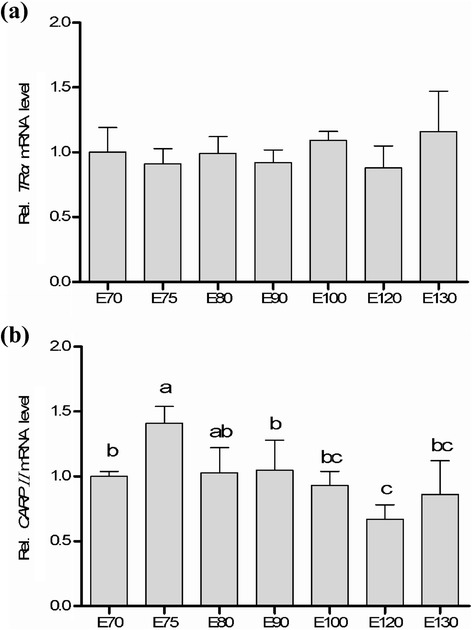
The quantitative expressions of *TRα* (**a**) and *CRABPII* mRNAs (**b**) in skin tissue of Inner Mongolian Cashmere goat. The bar height presented the means, and error bar displayed +1SE (*n* = 3). Different letters above the bars indicate a significant difference (*P* < 0.05) between different stages

To better understand the prenatal dynamical expressions of *TRα* and *CRABPII* in the skin tissue of cashmere goats, the qRT-PCR array was performed in the middle late embryonic stages (E70 to E130). As shown in Fig. 3, both of *TRα* and *CRABPII* mRNAs were detectable in all the tested time points. However, no significant difference of *TRα* gene expression was detected during the middle late development of goat embryos. The mRNA of *TRα* was expressed at E70 with relatively high level and mildly decreased in the following three stages (E75, E80, and E90), and then increased at E100 and reduced to the lowest level at E120, subsequently. The highest expression of *TRα* gene was observed in the last stage (E130, *P* > 0.05). The previous studies has reported that the secondary follicles grew from E65 to E75 and then extended to skin surface. The complete structure of the secondary follicle was formed at E135 in Chinese cashmere goats [4, 28, 29]. Synchronously coupled with the early formation and growth of cashmere, the mRNA expression of *TRα* gene was up-regulated indicating that *TRα* could play a role in the time-course growth of goat cashmere.

The expression pattern of *CRABPII* mRNA showed an “up-down-up” trend, which revealed a significantly highest expression at E75 (*P* < 0.05), and was down-regulated during E80 to E120 (*P* < 0.05) then increased again at E130. In embryonic development of hair follicles, the glandula sebacea cells were observed in the skin tissue from cashmere goat fetus at E85 [30]. The glandula sebacea formed at E90 and accelerated the growth of primary hair follicles. However, the physiologic difference between primary and secondary follicles was that no glandula sebacea was found in secondary hair follicles. The second hair follicles grew retard and partially matured at E130. The mRNA expression of *CRABPII* at E90 was lower than that at E80 when no glandula sebacea was formed. The *CRABPII* gene could regulate the early development of glandula sebacea though modifying the concentration of RA. The mRNA of *CRABPII* gene at E100 expressed significantly higher than that at E120, which led more RA transported into nucleus and bound to its receptor, and proposed to boost the growth of glandula sebacea. In humans, the concentration of RA in cells could increase the mRNA expression of *CRABPII* in skin [31, 32].

In this study, we characterized the caprine *TRα and CRABPII* genes and quantified their mRNA expressions during the formation of secondary hair follicles in the middle late embryonic periods. Our study will enrich the knowledge of goat *TRα* and *CRABPII* genes and provide the foundation for further insight into their functions on cashmere growth.

## Conclusions

Cashmere wool is the very valuable production obtained from goats. It is very important to investigate the expressions of key functional genes associated with cashmere growth during the prenatal and process-oriented periods (from anagen to catagen and finally telogen). Taken together, our results profiled the expressions of TRα and CRABPII genes associated with prenatal development of goat hair follicle.

## Additional files

Additional file 1: Figure S1. Alignment of the *TRα* coding sequences in mammals (PPT 230 kb) Additional file 2: Figure S2. Alignment of the *CRABPII* nucleotide sequences in mammals (PPT 146 kb) Additional file 3: Table S1. The sequence similarity of *TRα* and *CRABPII* genes in this study (DOC 39 kb)
