# Supplementary figures and images for: Expression patterns of TRα and CRABPII genes in Chinese cashmere goat skin during prenatal development
Source: J Anim Sci Technol. 2015 Aug 20;57:28. doi: 10.1186/s40781-015-0060-6 (PMC4940992; doi:10.1186/s40781-015-0060-6)

## Slide 1
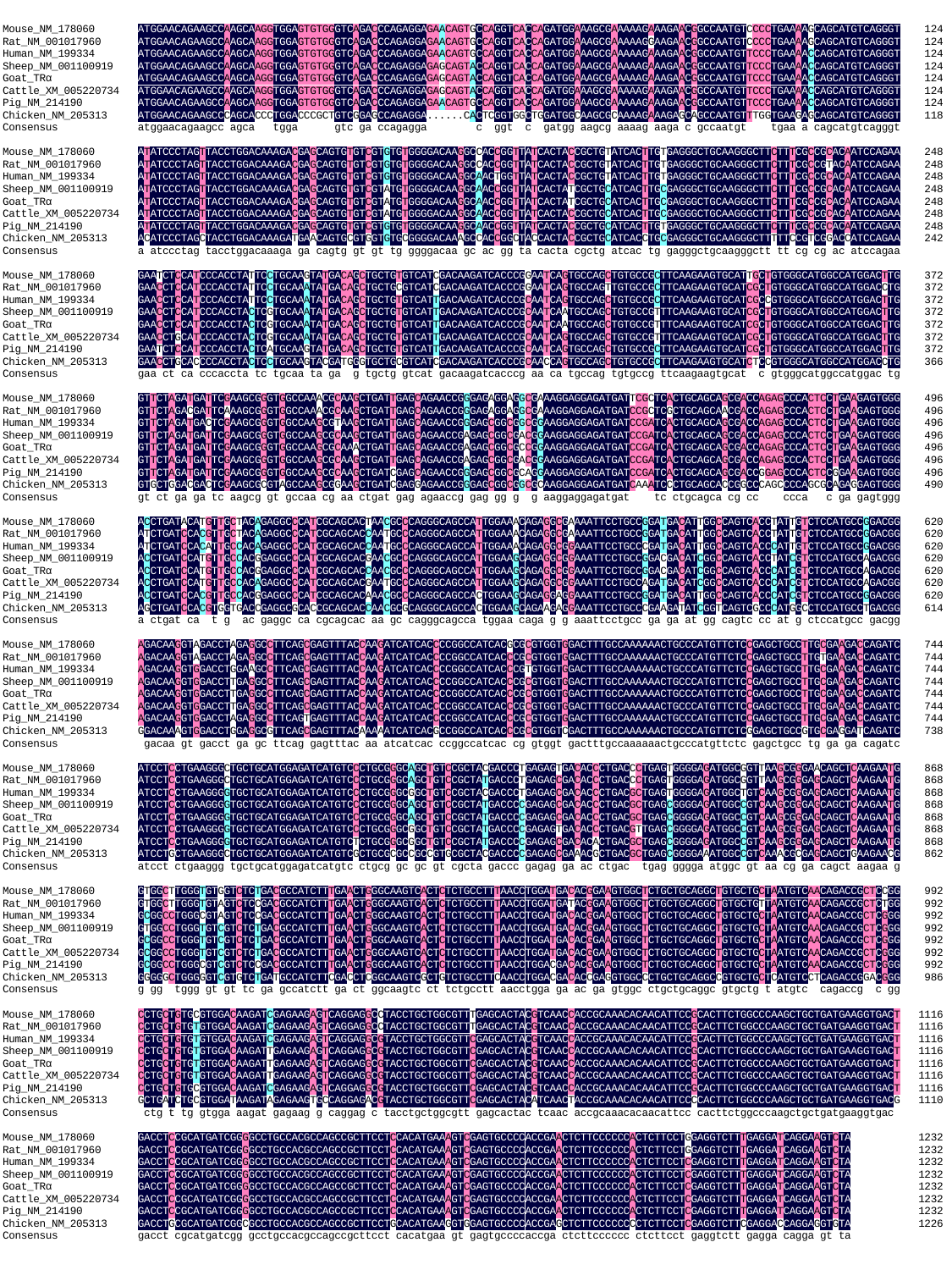

Supplement: Additional file 1: Figure S1. — Alignment of the TRα coding sequences in mammals (PPT 230 kb) [file 40781_2015_60_MOESM1_ESM.ppt]

## Slide 1
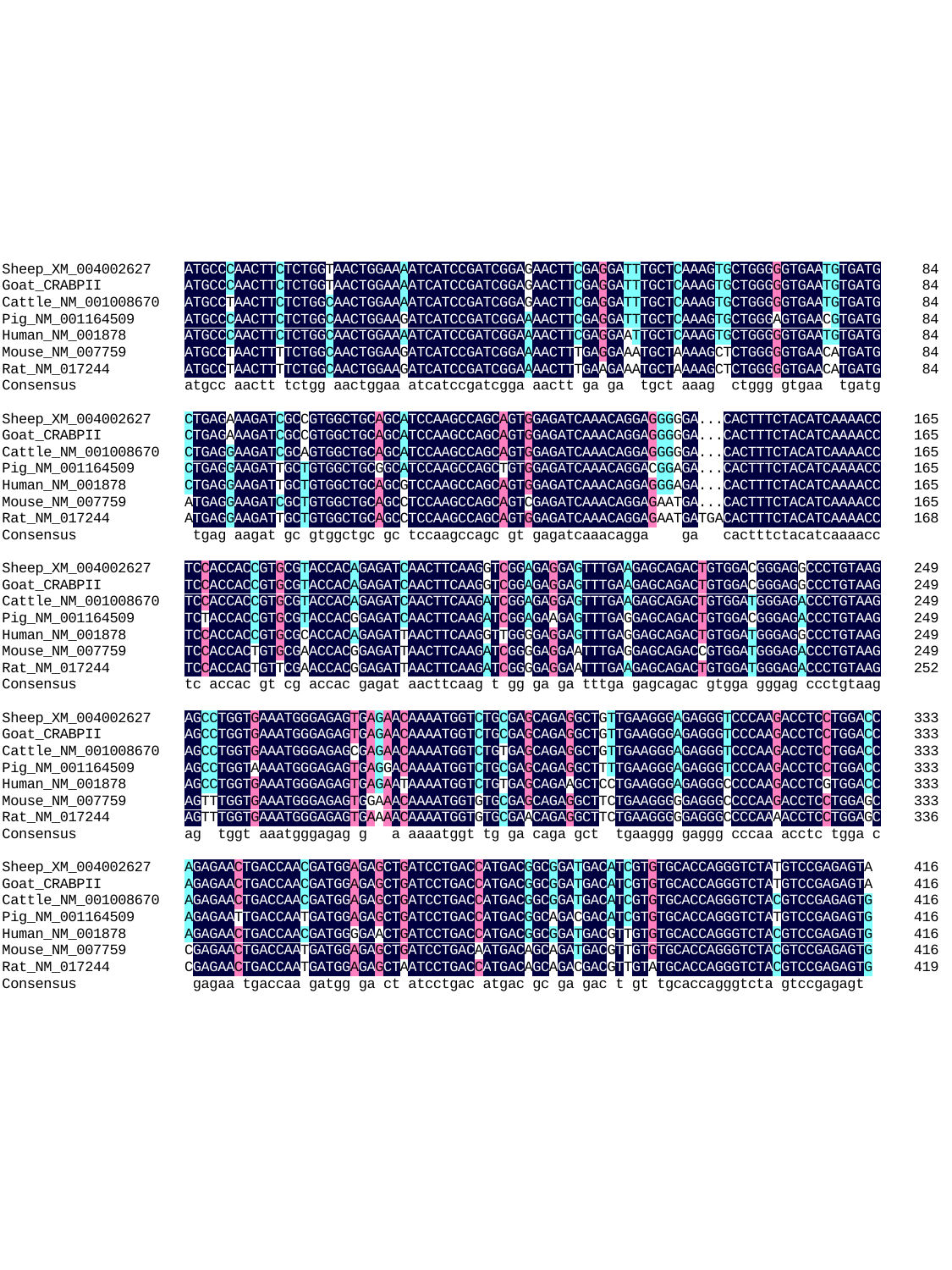

Supplement: Additional file 2: Figure S2. — Alignment of the CRABPII nucleotide sequences in mammals (PPT 146 kb) [file 40781_2015_60_MOESM2_ESM.ppt]
