# Supplementary material for: Expression patterns of TRα and CRABPII genes in Chinese cashmere goat skin during prenatal development
Source: J Anim Sci Technol. 2015 Aug 20;57:28. doi: 10.1186/s40781-015-0060-6 (PMC4940992; doi:10.1186/s40781-015-0060-6)
Supplement: Additional file 3: Table S1. — The sequence similarity of TRα and CRABPII genes in this study (DOC 39 kb) [file 40781_2015_60_MOESM3_ESM.doc]

**Table 1** The RT-PCR and real-time PCR primers used in this study

| Primer Name | Sequence (5′-3′) | Fragment  size (bp) | T.M. (°C) |
| --- | --- | --- | --- |
| *Cloning primers* | | | |
| *TRα-*1F | CCTGGATGGAATTGAAGTGA | 799 | 62.0 |
| *TRα-*1R | GACATGATCTCCATGCAGC |  |  |
| *TRα-*2F | AGGCCTTCAGCGAGTTTAC | 652 | 59.0 |
| *TRα-*2R | CCTTCTCTCCAGGCTCCTC |  |  |
| *CRABPII*-1F | CAGTGCTCCAGTGGAAAGA | 563 | 56.5 |
| *CRABPII*-1R | CCAGAAGTGATTGGGTGAG |  |  |
| *Real-time PCR primers* | | | |
| *TRα-*3F | TTACCTGGACAAAGACGAGC | 113 | 57.4 |
| *TRα-*3R | TCTGGATTGTGCGGCGAAAG |  |  |
| *CRABPII*-2F | ACATCAAAACCTCCACCACC | 111 | 56.5 |
| *CRABPII*-2R | CCCATTTCACCAGGCTCTTA |  |  |
| *ACTB-*F | CCTGCGGCATTCACGAAACTAC | 87 | 58.5 |
| *ACTB-*R | ACAGCACCGTGTTGGCGTAGAG |  |  |
| *GAPDH-*F  *GAPDH-*R  *TOP2B-*F  *TOP2B-*R | GCA AGTTCCACGGCACAG  GGT TCACGCCCATCACAA  GTGTGGAGCCTGAGTGGTATA  AAGCATTCGCCTGACATTGTT | 249  137 | 59.0  59.0 |
